# A Phase I study of intravenous PI3K inhibitor copanlisib in Japanese patients with advanced or refractory solid tumors

**DOI:** 10.1007/s00280-016-3198-0

**Published:** 2016-12-03

**Authors:** Toshihiko Doi, Nozomu Fuse, Takayuki Yoshino, Takashi Kojima, Hideaki Bando, Hideaki Miyamoto, Masato Kaneko, Motonobu Osada, Atsushi Ohtsu

**Affiliations:** 1 National Cancer Center Hospital East, 5-1, Kashiwanoha 6-chome, Kashiwa-shi, Chiba 277-8577 Japan; 2 Department of Gastroenterology and Hepatology, Kumamoto University, 2-39-1 Kurokami, Chuo Ward, Kumamoto, 860-8555 Japan; 3 Bayer Yakuhin, Ltd., 2-4-9, Umeda, Kita-ku, Osaka, 530-0001 Japan

**Keywords:** Copanlisib, PI3K inhibitor, Japanese, Solid tumors

## Abstract

**Purpose:**

To evaluate the safety, tolerability, pharmacokinetics, and efficacy of the intravenously administered pan-PI3K inhibitor copanlisib in Japanese patients with advanced or refractory solid tumors.

**Methods:**

A Phase I open-label study in Japanese patients with advanced or refractory solid tumors was carried out. Patients received a single intravenous dose of either copanlisib 0.4 mg/kg or copanlisib 0.8 mg/kg, dosed intermittently on days 1, 8, and 15 of a 28-day cycle. Safety was monitored throughout the study. Plasma copanlisib levels were measured for pharmacokinetic analysis.

**Results:**

Ten patients were enrolled and treated; three received copanlisib 0.4 mg/kg and seven received copanlisib 0.8 mg/kg. Overall, median duration of treatment was 6.2 weeks. No patients treated at 0.4 mg/kg experienced a dose-limiting toxicity, and the maximum tolerated dose in Japanese patients was determined to be 0.8 mg/kg. Adverse events were recorded in all ten patients; the most common were hyperglycemia, hypertension, and constipation. Copanlisib pharmacokinetic exposures displayed near dose-proportionality, with no accumulation. No patients achieved a complete or partial response, and disease control rate was 40.0%.

**Conclusions:**

Copanlisib was well tolerated in Japanese patients with advanced or refractory solid tumors, and the maximum tolerated dose was determined to be 0.8 mg/kg. Copanlisib demonstrated near dose-proportional pharmacokinetics and preliminary disease control, warranting further investigation.

***Clinical trial registration number*:**

NCT01404390.

## Introduction

The phosphatidylinositol 3-kinases (PI3Ks) are a family of lipid kinases with key roles in intracellular signaling cascades regulating many cellular processes [1]. However, PI3K-mediated activation of downstream effectors, including the serine/threonine kinase AKT and mammalian target of rapamycin (mTOR), is key to promoting cell survival proliferation and differentiation and is aberrantly activated in a variety of human cancers [2, 3]. Activation of the PI3K/AKT pathway is one of the major mechanisms by which tumors escape negative regulation of proliferation and become resistant to chemotherapy, targeted agents, and radiation. PI3K inhibitors therefore provide a promising therapeutic strategy not only in PI3K pathway-driven tumors but also in combination with other chemotherapy agents [4].

Copanlisib (BAY 80-6946; Bayer Pharma AG, Berlin, Germany) is a potent pan-class I PI3K inhibitor that is highly selective for PI3K compared with mTOR, and with preferential activity against the p110α and p110δ isoforms of PI3K, compared with the p110β and p110γ isoforms [5]. Copanlisib has demonstrated promising antitumor activity in preclinical tumor models with either *PIK3CA* mutation or *PTEN* deletion and/or overexpression of human epidermal growth factor receptor 2 [6]. Preclinical data from models of non-small-cell lung cancer, colorectal cancer, and breast cancer have provided support for the investigation of the combination of PI3K- and mitogen-activated protein kinase inhibition [7–9]. Copanlisib is therefore a potential candidate for use in PI3K-driven tumors either alone or in combination with other agents.

In a first-in-human Phase I study conducted in the USA, copanlisib was generally safe and well tolerated, and the maximum tolerated dose (MTD) was determined to be 0.8 mg/kg [10]. Copanlisib also showed promising antitumor pharmacodynamic activity and preliminary clinical benefit in patients with a range of advanced solid tumors and in patients with non-Hodgkin’s lymphoma. This study evaluated the safety, tolerability, pharmacokinetics (PK), and efficacy of copanlisib in Japanese patients with advanced or refractory solid tumors.

## Materials and methods

This study was conducted in accordance with the Declaration of Helsinki. Documented approval from the appropriate ethics committees and institutional review boards was obtained for all participating centers before study initiation, where required.

### Study design

This uncontrolled, open-label, non-randomized, single-center, Phase I study evaluated the safety, tolerability, and PK as a primary objective, and the efficacy as a secondary objective, of copanlisib in Japanese patients with advanced or refractory solid tumors. The study comprised two cohorts, one at a dose of copanlisib 0.4 mg/kg (cohort 1) and one at copanlisib 0.8 mg/kg (cohort 2), the MTD determined in a previous first-in-human study [10]; this study aimed to evaluate whether the previously defined MTD in a non-Japanese study could be applied to Japanese patients with solid tumors.

Patients received a single intravenous infusion of copanlisib over 1 h on days 1, 8, and 15 of a 28-day cycle, with 1 week of rest. On cycle 1, day 1, patients were required to fast for at least 8 h before and for 2 h following the end of the copanlisib infusion, at which point food intake was permitted. Dosing on days 8 and 15 was permitted within a window of ±1 day if necessary, and dosing was considered missed if delayed by more than 1 day. Dose delays of up to 2 weeks were permitted for day 1 of cycle 2 or later, although for days 8 and 15 of cycle 2 and subsequent cycles, dosing within a window of ±3 days was permitted, except for in cycle 3, where dosing was to be administered within ±1 day of days 8 and 15.

Patients were enrolled into cohort 1 and were evaluated for the occurrence of a dose-limiting toxicity (DLT) as defined by the National Cancer Institute Common Terminology Criteria for Adverse Events version 4.03. If no DLT was observed in cycle 1, or if treatment was continued with dose reduction, and if patients were deemed to derive treatment benefit per the investigators’ judgment, then patients were either enrolled into cohort 2 (copanlisib 0.8 mg/kg) with additional informed consent or able to discontinue study treatment. Completion of cycle 1 in cohort 1 constituted study completion. During cycle 2, patients received copanlisib 0.8 mg/kg and treatment was continued until disease progression or unacceptable toxicity.

Three patients were to be initially enrolled into cohort 1; if no DLT was observed, cohort 2 was to be initiated. If a DLT was observed in one of the three patients in cohort 1, an additional three patients were to be enrolled into cohort 1. If a DLT was observed in one of the six patients, cohort 2 was to be initiated. Enrollment to cohort 1 was discontinued if a DLT was observed in two or more patients, and subsequent enrollment was to be determined by the sponsor following consultation with the investigator. Six patients were to be treated in cohort 2, with dose acceptability confirmed following consultation with the investigator.

Protocol-defined DLTs included non-hematologic adverse events of grade ≥3. Hyperglycemia defined as a DLT was to be determined based on serum glucose values. Hematologic DLTs were defined as grade 4 neutropenia for ≥3 days, an absolute neutrophil count <1000 with fever ≥38.5 °C, and grade 4 thrombocytopenia. Patients with non-hematologic DLTs were to discontinue study treatment. However, patients with grade 3 hyperglycemia based on a fast of ≥8 h who responded to insulin, or hyperglycemia that became grade ≤2 within 24 h of insulin administration, and patients dosed at 0.8 mg/kg and who experienced non-hematologic DLTs other than hyperglycemia, but showed subjective or objective clinical benefit, could continue the study at the reduced dose of 0.4 mg/kg.

Dose reduction from 0.8 to 0.4 mg/kg was permitted: following a non-glucose and non-hematologic DLT in the previous cycle, when dosing on days 8 and 15 of the previous cycle was missed because of hematologic toxicity or hematologic DLT; and when two or more cases of glucose-related DLT occurred in the previous cycle despite a change in insulin regimen. Patients who did not tolerate the 0.4 mg/kg dose were to be withdrawn from the study.

Patients were hospitalized for safety observations during cycle 1, after which patients could be discharged at the investigators’ discretion. In cohort 1, the second patient was not enrolled until completion of the safety assessment following the first copanlisib infusion in the first patient.

### Patients

Japanese patients at least 20 years old with advanced or refractory, histologically or cytologically confirmed, non-hematologic, malignant solid tumors unamenable to standard therapy (without past or current central nervous system involvement) were eligible for inclusion in this study. Additional inclusion criteria were: at least one measurable lesion or evaluable disease according to Response Evaluation Criteria in Solid Tumors (RECIST) version 1.1; Eastern Cooperative Oncology Group performance status (ECOG PS) 0 or 1; and life expectancy >12 weeks. Patients were excluded if they met the following criteria: investigational drug therapy, anticancer chemotherapy, immunotherapy, or radiotherapy to target lesions within 4 weeks before study entry; type 1 or 2 diabetes mellitus or a fasting blood glucose level >125 mg/dL at screening and/or glycated hemoglobin ≥6.5%; past or current history of cardiac disease or congestive heart failure (New York Heart Association Class II); active coronary artery disease; myocardial infarction within 6 months of study entry; or new onset of angina <3 months before study entry. Additional exclusion criteria included: active and serious infections of grade >2 as defined by National Cancer Institute Common Terminology Criteria for Adverse Events version 4.03; seizure disorders requiring medication; uncontrolled hypertension (defined as systolic blood pressure >150 mmHg or diastolic pressure >90 mmHg, despite optimal management); known HIV infection or chronic hepatitis B or C virus infection; inadequate bone marrow, liver, and renal functions (as assessed by laboratory values of hemoglobin <9.0 g/dL, absolute neutrophil count <1500/mm^3^, platelet count <100,000/mm^3^, total bilirubin >2 × upper limit of normal [ULN], alanine aminotransferase and aspartate aminotransferase >2.5 × ULN [or >5 × ULN for patients with liver involvement in their cancer], serum creatinine >1.5 × ULN, creatinine clearance ≥50 mL/min, prothrombin time/prothrombin time international normalized ratio ≥1.5 × ULN); renal dialysis; bleeding diathesis; ongoing substance abuse; pregnant or breastfeeding; and the use of strong inhibitors of CYP3A4.

### Assessments

Screening comprised: provision of written, informed consent; demographics; baseline characteristics; concomitant medications; safety assessments (adverse events [defined as any new finding not present in the patient’s medical history, or a worsening of a prior medical history finding], vital signs, laboratory tests, physical examination, 12-lead electrocardiogram, multigated acquisition scan, or echocardiogram); tumor measurements by computed tomography or magnetic resonance imaging; disease assessment as defined by RECIST version 1.1; ophthalmologic examination; chest computed tomography; and ECOG PS assessment. The following assessments were also performed on cycle 1, day 1: safety; ECOG PS assessment; concomitant medications; blood samples for PK analysis; serum samples for glucose analysis; serum insulin; urinalysis; serum ketones; and post-dose echocardiogram. Safety assessments, blood pressure measurements, blood sampling for PK analysis, and urinalysis were also performed on days 8 and 15 of cycle 1.

Blood sampling for PK analysis of serum was performed on cycle 1, day 1 at 0, 0.5, 1, 1.5, 2, 3, 5, 8, 11, 25, 49, and 168 h following the start of copanlisib infusion. Additional PK samples were collected at either 72, 96, or 120 h following the start of infusion. PK samples were also collected on cycle 1, day 15 at 0, 0.5, 1, 1.5, 2, 3, 5, 8, 11, and 25 h following the start of infusion and on cycle 3, day 15 at 0, 0.5, 1, 1.5, 2, 3, 5, and 8 h following the start of infusion. Blood pressure monitoring was performed every 5 min before dosing on cycle 1, day 1 until the observation of two consecutive measurements <150 mmHg (systolic) and <90 mmHg (diastolic). Blood pressure was also measured before the collection of each blood PK sample, up to 49 h following the start of copanlisib infusion. Serum samples for glucose analysis were collected immediately before the first infusion and in parallel with PK sampling through to 49 h post-dose. Glucose samples taken at 25 h post-dose were to be fasting, where possible and if deemed safe. Capillary blood glucose testing was to be performed for hyperglycemia management as determined appropriate by the investigator; hyperglycemia was defined as capillary blood glucose >200 mg/dL and was to be treated with short-acting insulin. Tumor measurement and disease assessment according to RECIST version 1.1 were conducted every two cycles by the investigator.

### Statistical analysis

This study was primarily a descriptive safety and tolerability trial, and no formal sample-size estimation was performed. Summary statistics were used for demographics, baseline characteristics, and PK parameters. Frequency tables were used for categorical variables.

## Results

### Baseline demographics and characteristics

Ten patients were enrolled into the study and all received treatment. Three patients received copanlisib 0.4 mg/kg and seven patients received copanlisib 0.8 mg/kg. The majority of patients were female (60.0%), with a median age of 59.5 years (range 51.0–65.0) (Table 1). The majority of patients had ECOG PS 0 at baseline (80.0%), and colon cancer was the most common tumor type (40.0%). Median time since initial diagnosis was 109.9 weeks for patients in cohort 1 and 190.4 weeks for patients in cohort 2 (Table 1). All patients had received prior systemic anticancer therapy (Table 1).

**Table 1:** Demographics and baseline characteristics

	Cohort 1, 0.4 mg/kg (*n* = 3)	Cohort 2, 0.8 mg/kg (*n* = 7)	Total (*N* = 10)
Female, *n* (%)	3 (100)	3 (42.9)	6 (60.0)
Asian race, *n* (%)	3 (100)	7 (100)	10 (100)
Median age, years (range)	59.0 (57.0–64.0)	60.0 (51.0–65.0)	59.5 (51.0–65.0)
*Eastern Cooperative Oncology Group performance status, n* (%)			
0	3 (100)	5 (71.4)	8 (80.0)
1	0	2 (28.6)	2 (20.0)
*Cancer classification*, *n* (%)			
Non-small-cell lung	0	2 (28.6)	2 (20.0)
Bladder	0	1 (14.3)	1 (10.0)
Kidney	1 (33.3)	0	1 (10.0)
Colon	2 (66.7)	2 (28.6)	4 (40.0)
Pancreatic adenocarcinoma	0	1 (14.3)	1 (10.0)
Gastrointestinal stromal tumor	0	1 (14.3)	1 (10.0)
Median time since initial diagnosis, weeks (range)	109.9 (98.9–153.1)	190.4 (27.0–548.3)	131.5 (27.0–548.3)
Prior systemic anticancer therapy, *n* (%)	3 (100)	7 (100)	10 (100)
*Number of prior systemic anticancer therapies*, *n* (%)			
1	1 (33.3)	1 (14.3)	2 (20.0)
2	1 (33.3)	0	1 (10.0)
3	1 (33.3)	1 (14.3)	2 (20.0)
4	0	2 (28.6)	2 (20.0)
5	0	1 (14.3)	1 (10.0)
6	0	2 (28.6)	2 (20.0)

### Copanlisib treatment

Overall, the median duration of treatment was 6.2 weeks (range 1.1–21.1): 6.3 weeks (range 6.1–10.9) in cohort 1 and 6.1 weeks (range 1.1–21.1) in cohort 2. The median number of treatment cycles, including interruptions, delays, and drug holidays, was two overall (range 1–6): two cycles (range 2–3) in cohort 1 and two cycles (range 1–6) in cohort 2. The median number of copanlisib infusions received was six (range 2–15) across both cohorts, with a range of 6–8 infusions in cohort 1 and a range of 2–15 infusions in cohort 2.

No patients experienced DLTs in cohort 1. A non-hematologic DLT (grade 3 diarrhea, which was recorded as possibly related to the study drug) was recorded in one patient receiving copanlisib 0.8 mg/kg in cohort 2. The patient with the DLT was withdrawn from the study and recovered, and no further DLTs were reported. The MTD in Japanese patients was therefore determined to be copanlisib 0.8 mg/kg. One patient in cohort 2 did not receive the third dose of copanlisib in cycle 1 as planned because of disease progression, and discontinued the study, prompting enrollment of one additional patient into this cohort.

### Safety

All ten patients experienced at least one treatment-emergent adverse event (TEAE), and all experienced at least one adverse event assessed by the investigator as drug-related. The most commonly reported TEAEs of all grades irrespective of causality were hyperglycemia (80.0%), hypertension (70.0%), and constipation (50.0%) (Table 2). TEAEs of grade 3 as worst grade were reported in five patients overall (50.0%), with two considered drug-related (20.0%) (Table 2). Grade 3 or 4 TEAEs were reported in one patient from cohort 1 (33.3%) and five patients from cohort 2 (71.4%) (Table 3). Grade 3 hyperglycemia, diarrhea, and lymphocytopenia were considered to be possibly drug-related (Table 3). One grade 4 TEAE was recorded and was not considered to be drug-related: increased aspartate aminotransferase in one patient with liver metastases in cohort 2. Serious adverse events were reported in three patients in cohort 2: grade 2 presyncope and grade 3 diarrhea in one patient (both events considered drug-related), grade 3 hepatobiliary disorders (not considered drug-related) in one patient, and grade 3 stroke (not considered drug-related) in another. No grade 5 adverse events were reported, irrespective of causality.

**Table 2:** Summary of adverse events

*n* (%)	Cohort 1, 0.4 mg/kg (*n* = 3)	Cohort 2, 0.8 mg/kg (*n* = 7)	Total (*N* = 10)
Any AE	3 (100)	7 (100)	10 (100)
Grade 3 AE	1 (33.3)	4 (57.1)	5 (50.0)
Grade 4 AE	0	1 (14.3)	1 (10.0)
Any serious AE	0	3 (42.9)	3 (30.0)
AEs leading to dose modification	1 (33.3)	5 (71.4)	6 (60.0)
AEs leading to permanent discontinuation of study drug	0	2 (28.6)	2 (20.0)
Any drug-related AE	3 (100)	7 (100)	10 (100)
Grade 3 drug-related AE	0	2 (28.6)	2 (20.0)
*Treatment*-*emergent AEs of all grades, irrespective of causality, occurring in* ≥*2 patients in total*			
Hyperglycemia	1 (33.3)	7 (100)	8 (80.0)
Hypertension	1 (33.3)	6 (85.7)	7 (70.0)
Constipation	3 (100)	2 (28.6)	5 (50.0)
Lymphocytopenia	0	4 (57.1)	4 (40.0)
Increased ALP	1 (33.3)	3 (42.9)	4 (40.0)
Neutropenia	1 (33.3)	3 (42.9)	4 (40.0)
Tumor pain	1 (33.3)	3 (42.9)	4 (40.0)
Leukopenia	1 (33.3)	3 (42.9)	4 (40.0)
Fever	2 (66.7)	2 (28.6)	4 (40.0)
Investigations, other	2 (66.7)	2 (28.6)	4 (40.0)
Anemia	0	3 (42.9)	3 (30.0)
Anorexia	0	3 (42.9)	3 (30.0)
Nausea	0	3 (42.9)	3 (30.0)
Acneiform rash	1 (33.3)	2 (28.6)	3 (30.0)
Increased ALT	1 (33.3)	2 (28.6)	3 (30.0)
Increased AST	0	2 (28.6)	2 (20.0)
Flu-like symptoms	0	2 (28.6)	2 (20.0)
Insomnia	0	2 (28.6)	2 (20.0)
Malaise	0	2 (28.6)	2 (20.0)
Oral mucositis	0	2 (28.6)	2 (20.0)
Pruritus	0	2 (28.6)	2 (20.0)
Cough	1 (33.3)	1 (14.3)	2 (20.0)

*AE* adverse event, *ALP* alkaline phosphatase, *ALT* alanine aminotransferase, *AST* aspartate aminotransferase

**Table 3:** Grade 3 or 4 treatment-emergent adverse events and investigator assessment of relationship to study drug

*n* (%)	Grade	Cohort 1, 0.4 mg/kg (*n* = 3)	Cohort 2, 0.8 mg/kg (*n* = 7)	Relationship to study drug
Increased AST	4	0	1 (14.3)	Not likely to be related
Increased ALP	3	1 (33.3)	1 (14.3)	Not likely to be related
Increased ALT	3	0	2 (28.6)	Not likely to be related
Anemia	3	0	2 (28.6)	Not likely to be related
Hyperglycemia	3	0	1 (14.3)	Possibly related
Diarrhea	3	0	1 (14.3)	Possibly related
Hepatobiliary disorder	3	0	1 (14.3)	Not likely to be related
Insomnia	3	0	1 (14.3)	Not likely to be related
Lymphocytopenia	3	0	1 (14.3)	Possibly related
Stroke	3	0	1 (14.3)	Not likely to be related

*ALP* alkaline phosphatase, *ALT* alanine aminotransferase, *AST* aspartate aminotransferase

All patients reported at least one hematologic and biochemical toxicity event, the most frequent of which were increased creatinine and hyperglycemia (both in all ten patients), hypoalbuminemia and anemia (90.0% overall; two patients in cohort 1 and seven patients in cohort 2), and lymphocytopenia (90% overall; three patients in cohort 1 and six in cohort 2).

Two adverse events leading to study discontinuation were reported (20.0%), both occurring in cohort 2 (Table 2). These were grade 3 diarrhea in one patient and grade 1 thrombocytopenia in another; both events were considered drug-related. Overall, no intra-cycle dose modification or dose modification for subsequent cycles was performed.

Eight patients withdrew from the study because of disease progression (three in cohort 1 and five in cohort 2): two patients discontinued in cycle 1, five in cycle 2, two in cycle 3, and one in cycle 5.

### Pharmacokinetics

**Fig. 1:**
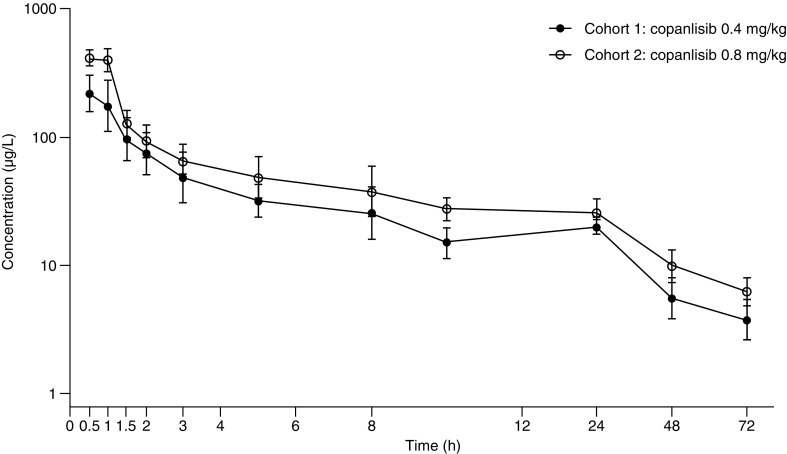
Geometric mean (geometric standard deviation) of copanlisib concentrations in plasma on cycle 1, day 1. Error bars represent geometric standard deviation

All patients were included in the single-dose PK assessment of copanlisib. Plasma geometric mean concentrations of copanlisib following administration on cycle 1, day 1 are presented in Fig. 1. Maximum drug concentration (*C*_max_) and exposure (area under the curve from 0 to 25 h after dosing [AUC_(0–25)_]) increased in a roughly dose-proportional manner between 0.4 and 0.8 mg/kg. Inter-patient variability in copanlisib *C*_max_ and AUC_(0–25)_ on cycle 1, day 1 was low to moderate, with coefficient of variation values of 14.5–32.5% for *C*_max_ and 19.3–32.0% for AUC_(0–25)_. No accumulation was observed after multiple dosing at either dose level, 0.4 mg/kg (Table 4) or 0.8 mg/kg (Table 5). The geometric mean terminal half-life was approximately 32 h (Table 4) to 36 h (Table 5) after single dosing.

**Table 4:** Geometric mean (% coefficient of variation) single- and multiple-dose pharmacokinetic parameters of copanlisib 0.4 mg/kg in cycles 1 and 3

	Cycle 1, day 1 (*n* = 3)	Cycle 1, day 15 (*n* = 3)	Cycle 3, day 15 (*n* = 1)
AUC, µg·h/L	1400 (31.1)	N/C	N/C
AUC_(0–8)_, µg·h/L	483 (38.5)	292 (15.3)	289
AUC_(0–25)_, µg·h/L	789 (32.0)	536 (13.6)	N/C
AUC_(0–25)_/D, µg·h/L	0.0382 (36.6)	0.0264 (9.22)	N/C
AUC_(0–25)norm_, kg·h/L	1.99 (29.3)	1.37 (11.9)	N/C
*C*_max_, µg/L	215 (34.7)	133 (23.1)	109
*C*_max_/D, 1/L	0.0104 (30.0)	0.0065 (17.3)	0.0055
*C*_max,norm_, kg/L	0.543 (31.7)	0.341 (21.0)	0.282
${{{t}_{\text{max}}}^{\text{a}}, {\text {h}}}$	0.50 [0.48–0.58]	0.50 [0.47–0.50]	1.02
*t*_1/2_, h	32.2 (76.8)	N/C	N/C

*AUC* area under the curve, *AUC*_*(0–8)*_ area under the curve from 0 to 8 h after dosing, *AUC*_*(0–25)*_ area under the curve from 0 to 25 h after dosing, *AUC*_*(0–25)*_ */D* *AUC*_*(0–25)*_ divided by dose (mg), *AUC*_*(0–25)norm*_ *AUC*_*(0–25)*_ divided by dose per kg body weight, *C*_*max*_ maximum drug concentration, *C*_*max,norm*_ maximum drug concentration divided by dose per kg body weight, *N/C* not calculated, *t*_*max*_ time to maximum drug concentration, *t*_*1/2*_ half-life associated with terminal slope

^a^Median [range]

**Table 5:** Geometric mean (% coefficient of variation) single- and multiple-dose pharmacokinetic parameters of copanlisib 0.8 mg/kg in cycles 1 and 3

	Cycle 1, day 1 (*n* = 7)	Cycle 1, day 15 (*n* = 6)	Cycle 3, day 15 (*n* = 1)
AUC, µg·h/L	2350 (14.3)	N/C	N/C
AUC_(0–8)_, µg·h/L	812 (17.8)	768 (20.4)	570
AUC_(0–25)_, µg·h/L	1280 (19.3)	1290 (10.1)	N/C
AUC_(0–25)_/D, µg·h/L	0.0274 (14.7)	0.0294 (10.8)	N/C
AUC_(0–25)norm_, kg·h/L	1.61 (18.6)	1.62 (11.1)	N/C
*C*_max_, µg/L	447 (14.5)	410 (37.0)	395
*C*_max_/D, 1/L	0.0096 (20.2)	0.0094 (31.3)	0.0084
*C*_max,norm_, kg/L	0.565 (13.9)	0.517 (38.0)	0.499
${{{t}_{\text{max}}}^{\text{a}}, {\text {h}}}$	0.53 [0.50-1.08]	0.75 [0.48-1.05]	1.02
t_1/2_, h	35.6 (73.1)	N/C	N/C

*AUC* area under the curve, *AUC*_*(0–8)*_ area under the curve from 0 to 8 h after dosing, *AUC*_*(0–25)*_ area under the curve from 0 to 25 h after dosing, *AUC*_*(0–25)*_ */D* *AUC*_*(0–25)*_ divided by dose (mg), *AUC*_*(0–25)norm*_ *AUC*_*(0–25)*_ divided by dose per kg body weight, *C*_*max*_ maximum drug concentration, *C*_*max,norm*_ maximum drug concentration divided by dose per kg body weight, *N/C* not calculated, *t*_*max*_ time to maximum drug concentration, *t*_*1/2*_ half-life associated with terminal slope

^a^Median [range]

### Treatment efficacy

All ten patients who received treatment were evaluable for efficacy. Of the ten patients, none experienced a complete or partial response. Four patients had stable disease (40.0%; one patient in cohort 1 and three in cohort 2) as best response, and six had progressive disease (60.0%; two patients in cohort 1 and four in cohort 2) as best response (Table 6). The disease control rate was therefore 40.0% (33.3% for cohort 1 and 42.9% for cohort 2).

**Table 6:** Best overall disease response with respect to Response Evaluation Criteria in Solid Tumors

*n* (%)	Cohort 1, 0.4 mg/kg (*n* = 3)	Cohort 2, 0.8 mg/kg (*n* = 7)	Total (*n* = 10)
Complete response	0	0	0
Partial response	0	0	0
Stable disease	1 (33.3)	3 (42.9)	4 (40.0)
Progressive disease^a^	2 (66.7)	4 (57.1)	6 (60.0)
Overall response rate (complete response + partial response)	0	0	0
Overall disease control rate (complete response + partial response + stable disease)	1 (33.3)	3 (42.9)	4 (40.0)

^a^By proven measurement

Overall, median time to progression was 52 days (95% confidence interval [CI] 21–82): 52 days (95% CI 50–77) for cohort 1 and 53 days (95% CI 21–not estimated) for cohort 2. In cohort 2, one patient with gastrointestinal stromal tumor had continued stable disease until the end of cycle 5 but discontinued treatment because of drug-related thrombocytopenia. Another patient in cohort 2 with stable disease discontinued treatment because of grade 3 diarrhea. Time to progression for these patients was censored at 176 and 50 days, respectively. Another two patients with stable disease as best response had a time to progression of 77 days (one patient in cohort 1) and 82 days (one patient in cohort 2). Overall, the rates of progression-free survival were 0.80 (95% CI 0.41–0.95) after 1 month, 0.35 (95% CI 0.08–0.64) after 2 months, and 0.12 (95% CI 0.01–0.40) after 3 months.

## Discussion

This Phase I study evaluated the safety, tolerability, PK, and efficacy of the pan-PI3K inhibitor copanlisib in Japanese patients with advanced or refractory solid tumors. Copanlisib was administered as a 1-h intravenous infusion once weekly for 3 weeks on days 1, 8, and 15 of a 28-day cycle.

The baseline demographics and disease characteristics of patients in this study were generally similar to those in the previously reported first-in-human study [10], except that patients in the current study were of Asian ethnicity and the current study did not include patients with hematologic malignancies. The starting dose used here (copanlisib 0.4 mg/kg once weekly) was determined as half of the MTD defined in the first-in-human study (copanlisib 0.8 mg/kg once weekly). Toxicities were expected to be manageable at the 0.4 mg/kg dose level with the use of short-acting insulin permitted to manage hyperglycemia, as reported in the first-in-human study [10]. The study design also ensured safety as patients were hospitalized for the duration of the first treatment cycle.

Copanlisib was well tolerated at the 0.4 mg/kg dose level, prompting enrollment at the 0.8 mg/kg dose level. Only one non-hematologic DLT was reported at the 0.8 mg/kg dose level, prompting enrollment of one further patient into cohort 2 to confirm safety at this dose level, and no further DLTs were observed; the MTD in Japanese patients was therefore determined to be 0.8 mg/kg once weekly and generally consistent with the preceding first-in-human study in non-Japanese patients [10]. Hyperglycemia and hypertension were the most commonly reported TEAEs, consistent with the first-in-human study [10]. Hyperglycemia is a known class effect of agents targeting the PI3K/AKT/mTOR signaling pathway [11] and was expected based on previous reports of other PI3K inhibitors [11–17] and the first-in-human study of copanlisib [10]. The hyperglycemia observed following copanlisib infusion is likely caused by inhibition of insulin signaling [18] and systemic insulin resistance [19], as previously reported in preclinical models of other PI3K/AKT pathway inhibitors. The mechanism for hypertension in this study is unclear and may arise from copanlisib-mediated inhibition of PI3K at the endothelial level leading to vasoconstriction [20], or through insulin-dependent vasoconstriction [21] as an effect of the post-infusion hyperglycemia, or both. However, the clinical significance of the increases in blood pressure following copanlisib infusion remains unclear. Hyperglycemia and hypertension events were all grade 1 or 2, except for one case of grade 3 hyperglycemia. These events were generally transient and manageable and did not lead to discontinuation in any patient. In the larger Phase I first-in-human study [10], hyperglycemia and hypertension were shown to be transient in nature, peaking at 5–8 or 1–2 h, respectively, after the start of infusion and then returning to normal values. The incidence of all-grade hypertension may have been higher here compared with the Patnaik et al. study (70 vs. 30%, respectively), but there were no reports of grade 3 hypertension here, compared with 19% grade 3 hypertension in the Patnaik et al. study [10]. In the current study, two adverse events resulted in discontinuation and were attributed as drug-related, one of which (grade 3 diarrhea) resolved following discontinuation and one (grade 1 thrombocytopenia) was unresolved. There were no cases of colitis, as has been reported for other orally administered PI3K inhibitors [12, 15]. No deaths on study were reported.

Gastrointestinal toxicity was relatively infrequent and mild in nature. Five patients had constipation (four grade 1), three had nausea (two grade 1), and one had grade 3 diarrhea. Two patients had grade ≥3 elevated aminotransferase levels, and this incidence may have been higher than seen in the first-in-human study, where <10% of patients had grade ≥3 elevated aminotransferase levels [10]. The overall incidence and severity of gastrointestinal toxicity seen with copanlisib in the current study and in the first-in-human study are notably less than those reported for other orally administered PI3K inhibitors [12, 13, 15, 22, 23] and may be related to the route of administration and first-pass metabolism seen with orally administered drugs [10].

The PK exposures of copanlisib increased in near dose-proportional manner at 0.4 mg/kg and at the MTD, 0.8 mg/kg, consistent with dose escalation in the first-in-human study of copanlisib [10], with no accumulation and low to moderate inter-patient variability. The long plasma half-life of approximately 32–36 h suggests wide tissue distribution, supportive of once-weekly dosing, consistent with the first-in-human study [10]. Overall, copanlisib PK parameters appeared consistent between Japanese and non-Japanese patients; however, the sample sizes used in this study were small, and variability was observed in PK parameters, so these data should be interpreted with some caution. Recent analyses have indicated that an intermittent dosing schedule of 60 mg weekly on days 1, 8, and 15 of a 28-day cycle was likely to achieve a similar risk/benefit ratio as 0.8 mg/kg weight-based dosing [24], and the fixed-dose schedule is under evaluation in ongoing studies.

Efficacy was a secondary objective in this small study, and no complete or partial responses were observed. In the larger first-in-human study, there were three objective responses (one complete response and two partial responses; objective response rate of 12%) among 25 patients in a solid-tumor expansion cohort treated at the MTD [10]. The disease control rate (complete response, partial response, or stable disease) in Japanese patients was 40%, similar to that reported for patients in the advanced solid-tumor expansion cohort in the first-in-human study (44%) [10]. Copanlisib also showed promising antitumor activity in a small number of patients with non-Hodgkin’s lymphoma in the first-in-human study (seven out of nine patients) [10]. The preliminary efficacy data here remain inconclusive because of the exploratory nature of this analysis and the small number of patients evaluated in this study.

Overall, copanlisib was safe and well tolerated in Japanese patients with advanced or refractory solid tumors, administered on days 1, 8, and 15 of a 28-day treatment cycle. The most common adverse events were hyperglycemia and hypertension, both of which were transient in nature and did not result in discontinuation of treatment. Gastrointestinal toxicity was generally infrequent. The MTD of copanlisib in Japanese patients was defined as 0.8 mg/kg, and copanlisib PK data support a once-weekly dosing schedule. In this small study, copanlisib showed preliminary disease control, and these data support further investigation of the safety and efficacy of copanlisib in Japanese patients with solid tumors and other advanced malignancies. A Phase Ib study into the safety of copanlisib in Japanese patients with relapsed, indolent B-cell non-Hodgkin’s lymphoma is ongoing (NCT02342665), in addition to a wider program of studies into copanlisib safety, pharmacodynamics, PK, and efficacy in a range of patient populations and cancer types.

## References

[1] Steelman LS, Stadelman KM, Chappell WH, Horn S, Bäsecke J, Cervello M, Nicoletti F, Libra M, Stivala F, Martelli AM, McCubrey JA (2008). Akt as a therapeutic target in cancer. Expert Opin Ther Targets.

[2] Liu P, Cheng H, Roberts TM, Zhao JJ (2009). Targeting the phosphoinositide 3-kinase pathway in cancer. Nat Rev Drug Discov.

[3] Wong KK, Engelman JA, Cantley LC (2010). Targeting the PI3K signaling pathway in cancer. Curr Opin Genet Dev.

[4] Yuan TL, Cantley LC (2008). PI3K pathway alterations in cancer: variations on a theme. Oncogene.

[5] Haike K, Stasik E, Soujon M, Wengner AM, Petrova E, Genvresse I, Wilhelm S, Childs BH, Mumberg D, Liu N (2014) Molecular mechanisms supporting inhibition of PI3K isoforms by copanlisib in blocking B-cell signaling and tumor cell growth in diffuse large B-cell lymphoma. Poster 48 presented at the 1st American Society of Hematology Meeting on Lymphoma Biology, Colorado Springs, Colorado, USA

[6] Liu N, Rowley BR, Bull CO, Schneider C, Haegebarth A, Schatz CA, Fracasso PR, Wilkie DP, Hentemann M, Wilhelm SM, Scott WJ, Mumberg D, Ziegelbauer K (2013). BAY 80-6946 is a highly selective intravenous PI3K inhibitor with potent p110α and p110δ activities in tumor cell lines and xenograft models. Mol Cancer Ther.

[7] Engelman JA, Chen L, Tan X, Crosby K, Guimaraes AR, Upadhyay R, Maira M, McNamara K, Perera SA, Song Y, Chirieac LR, Kaur R, Lightbown A, Simendinger J, Li T, Padera RF, García-Echeverría C, Weissleder R, Mahmood U, Cantley LC, Wong KK (2008). Effective use of PI3K and MEK inhibitors to treat mutant *Kras* G12D and *PIK3CA* H1047R murine lung cancers. Nat Med.

[8] Lackner MR (2010). Prospects for personalized medicine with inhibitors targeting the RAS and PI3K pathways. Expert Rev Mol Diagn.

[9] O’Brien NA, McDonald K, Tong L, von Euw E, Kalous O, Conklin D, Hurvitz SA, di Tomaso E, Schnell C, Linnartz R, Finn RS, Hirawat S, Slamon DJ (2014). Targeting PI3K/mTOR overcomes resistance to HER2-targeted therapy independent of feedback activation of AKT. Clin Cancer Res.

[10] Patnaik A, Appleman LJ, Tolcher AW, Papadopoulos KP, Beeram M, Rasco DW, Weiss GJ, Sachdev JC, Chadha M, Fulk M, Ejadi S, Mountz JM, Lotze MT, Toledo FGS, Chu E, Jeffers M, Peña C, Xia C, Reif S, Genvresse I, Ramanathan RK (2016) First-in-human phase I study of copanlisib (BAY 80-6946), an intravenous pan-class I phosphatidylinositol 3-kinase inhibitor, in patients with advanced solid tumors and non-Hodgkin’s lymphomas. Ann Oncol 27:1928–1940 10.1093/annonc/mdw282 PMC5035790 27672108

[11] Busaidy NL, Farooki A, Dowlati A, Perentesis JP, Dancey JE, Doyle LA, Brell JM, Siu LL (2012). Management of metabolic effects associated with anticancer agents targeting the PI3K-Akt-mTOR pathway. J Clin Oncol.

[12] Gopal AK, Kahl BS, de Vos S, Wagner-Johnston ND, Schuster SJ, Jurczak WJ, Flinn IW, Flowers CR, Martin P, Viardot A, Blum KA, Goy AH, Davies AJ, Zinzani PL, Dreyling M, Johnson D, Miller LL, Holes L, Li D, Dansey RD, Godfrey WR, Salles GA (2014). PI3Kδ inhibition by idelalisib in patients with relapsed indolent lymphoma. N Engl J Med.

[13] Bendell JC, Rodon J, Burris HA, de Jonge M, Verweij J, Birle D, Demanse D, De Buck SS, Ru QC, Peters M, Goldbrunner M, Baselga J (2012). Phase I, dose-escalation study of BKM120, an oral pan-class I PI3K inhibitor, in patients with advanced solid tumors. J Clin Oncol.

[14] Shapiro GI, Bell-McGuinn KM, Molina JR, Bendell J, Spicer J, Kwak EL, Pandya SS, Millham R, Borzillo G, Pierce KJ, Han L, Houk BE, Gallo JD, Alsina M, Braña I, Tabernero J (2015). First-in-human study of PF-05212384 (PKI-587), a small-molecule, intravenous, dual inhibitor of PI3K and mTOR in patients with advanced cancer. Clin Cancer Res.

[15] Flinn IW, Kahl BS, Leonard JP, Furman RR, Brown JR, Byrd JC, Wagner-Johnston ND, Coutre SE, Benson DM, Peterman S, Cho Y, Webb HK, Johnson DM, Yu AS, Ulrich RG, Godfrey WR, Miller LL, Spurgeon SE (2014). Idelalisib, a selective inhibitor of phosphatidylinositol 3-kinase-δ, as therapy for previously treated indolent non-Hodgkin lymphoma. Blood.

[16] Yap TA, Yan L, Patnaik A, Fearen I, Olmos D, Papadopoulos K, Baird RD, Delgado L, Taylor A, Lupinacci L, Riisnaes R, Pope LL, Heaton SP, Thomas G, Garrett MD, Sullivan DM, de Bono JS, Tolcher AW (2011). First-in-man clinical trial of the oral pan-AKT inhibitor MK-2206 in patients with advanced solid tumors. J Clin Oncol.

[17] Britten CD, Adjei AA, Millham R, Houk BE, Borzillo G, Pierce K, Wainberg ZA, LoRusso PM (2014). Phase I study of PF-04691502, a small-molecule, oral, dual inhibitor of PI3K and mTOR, in patients with advanced cancer. Invest New Drugs.

[18] Knight ZA, Gonzalez B, Feldman ME, Zunder ER, Goldenberg DD, Williams O, Loewith R, Stokoe D, Balla A, Toth B, Balla T, Weiss WA, Williams RL, Shokat KM (2006). A pharmacological map of the PI3-K family defines a role for p110α in insulin signaling. Cell.

[19] Crouthamel MC, Kahana JA, Korenchuk S, Zhang SY, Sundaresan G, Eberwein DJ, Brown KK, Kumar R (2009). Mechanism and management of AKT inhibitor-induced hyperglycemia. Clin Cancer Res.

[20] Quaschning T, Voss F, Relle K, Kalk P, Vignon-Zellweger N, Pfab T, Bauer C, Theilig F, Bachmann S, Kraemer-Guth A, Wanner C, Theuring F, Galle J, Hocher B (2007). Lack of endothelial nitric oxide synthase promotes endothelin-induced hypertension: lessons from endothelin-1 transgenic/endothelial nitric oxide synthase knockout mice. J Am Soc Nephrol.

[21] Symons JD, McMillin SL, Riehle C, Tanner J, Palionyte M, Hillas E, Jones D, Cooksey RC, Birnbaum MJ, McClain DA, Zhang QJ, Gale D, Wilson LJ, Abel ED (2009). Contribution of insulin and Akt1 signaling to endothelial nitric oxide synthase in the regulation of endothelial function and blood pressure. Circ Res.

[22] Brown JR, Davids MS, Rodon J, Abrisqueta P, Kasar SN, Lager J, Jiang J, Egile C, Awan FT (2015). Phase I trial of the pan-PI3K inhibitor pilaralisib (SAR245408/XL147) in patients with chronic lymphocytic leukemia (CLL) or relapsed/refractory lymphoma. Clin Cancer Res.

[23] Sarker D, Ang JE, Baird R, Kristeleit R, Shah K, Moreno V, Clarke PA, Raynaud FI, Levy G, Ware JA, Mazina K, Lin R, Wu J, Fredrickson J, Spoerke JM, Lackner MR, Yan Y, Friedman LS, Kaye SB, Derynck MK, Workman P, de Bono JS (2015). First-in-human phase I study of pictilisib (GDC-0941), a potent pan-class I phosphatidylinositol-3-kinase (PI3K) inhibitor, in patients with advanced solid tumors. Clin Cancer Res.

[24] Reif S, Ahsman M, Jentsch G, Wiegert E, Grevel J, Granvil C (2016). Use of a population pharmacokinetic approach and time-to-event analysis to support the clinical recommendation of a flat dosing of copanlisib in cancer patients. Clin Pharmacol Ther.

